# Elevated Adaptive Immune Responses Are Associated with Latent Infections of *Wuchereria bancrofti*

**DOI:** 10.1371/journal.pntd.0001611

**Published:** 2012-04-03

**Authors:** Kathrin Arndts, Susanne Deininger, Sabine Specht, Ute Klarmann, Sabine Mand, Tomabu Adjobimey, Alexander Y. Debrah, Linda Batsa, Alexander Kwarteng, Christian Epp, Mark Taylor, Ohene Adjei, Laura E. Layland, Achim Hoerauf

**Affiliations:** 1 Institute of Medical Microbiology, Immunology and Parasitology, University Hospital Bonn, Bonn, Germany; 2 Institute of Medical Biometry, Informatics and Epidemiology, University Hospital Bonn, Bonn, Germany; 3 Kumasi Centre for Collaborative Research in Tropical Medicine, Kumasi, Ghana; 4 Faculty of Allied Health Sciences, Department of Theoretical and Applied Biology, and School of Medical Sciences, Kwame Nkrumah University of Science and Technology, Kumasi, Ghana; 5 Department für Infektiologie, Parasitologie, Universitätsklinikum Heidelberg, Heidelberg, Germany; 6 Liverpool School of Tropical Medicine, Liverpool, United Kingdom; University of Nottingham, United Kingdom

## Abstract

In order to guarantee the fulfillment of their complex lifecycle, adult filarial nematodes release millions of microfilariae (MF), which are taken up by mosquito vectors. The current strategy to eliminate lymphatic filariasis as a public health problem focuses upon interrupting this transmission through annual mass drug administration (MDA). It remains unclear however, how many rounds of MDA are required to achieve low enough levels of MF to cease transmission. Interestingly, with the development of further diagnostic tools a relatively neglected cohort of asymptomatic (non-lymphedema) amicrofilaremic (latent) individuals has become apparent. Indeed, epidemiological studies have suggested that there are equal numbers of patent (MF^+^) and latent individuals. Since the latter represent a roadblock for transmission, we studied differences in immune responses of infected asymptomatic male individuals (n = 159) presenting either patent (n = 92 MF^+^) or latent (n = 67 MF^−^) manifestations of *Wuchereria bancrofti*. These individuals were selected on the basis of MF, circulating filarial antigen in plasma and detectable worm nests. Immunological profiles of either Th1/Th17, Th2, regulatory or innate responses were determined after stimulation of freshly isolated PBMCs with either filarial-specific extract or bystander stimuli. In addition, levels of total and filarial-specific antibodies, both IgG subclasses and IgE, were ascertained from plasma. Results from these individuals were compared with those from 22 healthy volunteers from the same endemic area. Interestingly, we observed that in contrast to MF^+^ patients, latent infected individuals had lower numbers of worm nests and increased adaptive immune responses including antigen-specific IL-5. These data highlight the immunosuppressive status of MF^+^ individuals, regardless of age or clinical hydrocele and reveal immunological profiles associated with latency and immune-mediated suppression of parasite transmission.

## Author Summary

The tropical helminth infection lymphatic filariasis affects more than 120 million people worldwide and is considered a major public health concern. Over 90% of infections are elicited by *Wucheria bancrofti* and adult worms reside in the lymphatic system releasing millions of microfilariae (MF), which periodically circulate in the blood. New diagnostic tools have provided a method to determine asymptomatic patients that are amicrofilaremic: a subset of individuals that have so far been neglected but are of special interest since these patients represent a dead end in terms of parasite transmission. Therefore, we were interested in determining whether the absence of MF was associated with distinct immunological profiles and observed that indeed responses in MF^+^ patients were dampened. From the viewpoint of the helminth such overall suppression of immune responses may facilitate MF transmission. Latent individuals however, presented elevated filarial specific responses and extrapolating these findings to the host provides novel insight into possible protective mechanisms which either actively hinders the release of MF from worms or their travel to the periphery. Further research into these aspects may broaden the range of strategies currently employed to reduce transmission and in turn eliminate bancroftian filariasis.

## Introduction

Lymphatic filariasis (LF) is a tropical helminth disease that causes acute and chronic inflammation in patients spanning 72 countries. According to recent reports, an estimated 120 million people are infected with around 40 million seriously incapacitated and disfigured by the disease [1], [2]. The consequential socioeconomic impact has thus designated this infection a major public health concern. The infection is provoked by threadlike nematodes (*Brugia malayi*, *B. timori* or *W. bancrofti*), which are transmitted by anopheline and culicine mosquitoes. 91% of infections are caused by *W. bancrofti* [3] and adult worms release millions of microfilariae in periodical patterns that then circulate in the blood [1]. Although the detection of MF has been essential for diagnosing bancroftian filariasis, to verify asymptomatic latent infections other tests are required. For example, visualization of active nematodes (filarial dance sign, FDS) can be determined via ultrasonography of the lymphatic vessels in scrotal regions [4], [5]. The rapid antigen card test can also be employed since this test measures circulating filarial antigen (CFA) in the plasma [6], [7]. Interestingly, CFA screening has revealed that there are roughly equal proportions of MF^+^ and MF^−^ individuals and this latter group has remained largely undetected and neglected in previous studies since at the time of diagnosis affected individuals present no severe pathology [8]. Since circulating MF are indispensable in terms of transmission, studying the immunological profiles of latently infected individuals may provide essential information as to how MF are prevented from developing or traveling to the periphery and thus, for the development of new therapeutic strategies.

Although filarial infections are often chronic and persist over many years, the majority of patients elicit only few signs of disease [9]. Nevertheless, regardless of whether the individuals are microfilaremic or not, non-limited immune reactions can lead to different clinical manifestations such as lymphedema, urogenital disorders or hydroceles. Thus, the factor(s) responsible for eliciting the degree of clinical disease and pathology remain a matter of debate. However, the general consensus deems that alongside genetic traits these overt reactions are related to the intensity of the host's responses to dead or dying worms [1], [10], [11], [12], [13], [14] which are then possibly enhanced by stronger reactions to unrelated stimuli or secondary infections. In addition, the release of the bacterial endosymbiont *Wolbachia* from moribund larvae or adult worms may also be a factor especially since they trigger innate and Th1/Th17 adaptive responses [15], [16], [17], [18]. *Wolbachia* are essential for worm survival and this unique relationship has provided an alternative avenue for chemotherapeutic treatment [19], [20], [21].

Parasitic helminths are known to elicit dominant Th2 (IL-5, IL-13) responses whilst simultaneously inducing a suppressive milieu [22]. A key paradigm in filariasis is that patients with elevated levels of regulatory responses have high parasite numbers and low pathological symptoms whereas patients with few or no parasites and deliberating pathology mount strong filarial-specific responses [23], [24]. With regards to lymphatic filariasis, many studies have focused on the immunological differences between patients presenting different degrees of pathology [25], [26], [27]. For example, patently infected individuals with no clinical signs of disease are characterized by down-regulated IL-2 and IFN-γ responses with a shift towards Th2 (IL-4, IL-5) and Treg (IL-10 and TGF-β) responses: this milieu is thought to be helminth-mediated in order to evade host defenses and ensure helminth survival [9], [10], [28], [29]. In contrast, patients with chronic pathology display a stronger Th1 immune response [24], [29], [30] or even a Th17 response [31] which in turn induces the secretion of VEGF-C which is associated with the development of filarial lymphedema [20]. Pathological profiles of filarial-infected patients are also reflected in their Ig responses. For example, asymptomatic MF^+^ individuals present elevated IgG4 levels whereas those with chronic pathology have higher IgE∶IgG4 ratios [32], [33]. IgG4 is a non-complement fixating Ig that binds weakly to effector cell Fc receptors and can compete with IgE for antigen-binding sites [34], [35]. Its secretion from B cells is mediated by regulatory T cells in an IL-10 and TGF-β dependent manner [36], [37].

As mentioned above, with the introduction of concise diagnostic tools, field studies have elucidated that in comparison to MF^+^ individuals there are equal numbers of asymptomatic MF^−^ patients harboring cryptic infection [7]. Therefore, we deciphered the immunological profiles of 159 filarial-infected patients which were classified as patently (n = 92) or latently (n = 67) infected. On the basis of filarial-specific Ig levels in plasma or cytokine release following antigen-specific re-stimulation of PBMCs, this comprehensive study has determined the differences in adaptive immune responses between patient groups. Indeed, our data reveal that MF^+^ individuals are more strongly immune-suppressed than amicrofilaremic patients, providing novel insight into immune responses of MF^−^ patients who represent an impasse for the parasite's continuous transmission.

## Methods

### Study population and ethics statement

We studied a cohort population of 159 *W. bancrofti*-infected asymptomatic males (18–50 years) from an endemic region of Ahanta West District, Ghana, in 2008. Recruited individuals were part of a treatment study (ISRCTN15216778). Asymptomatic criteria was based on the lack of lymphedema; 12 patients did however show the presence of clinical hydrocele according to Mand *et al.* [38], (MF^+^ n = 7 and MF^−^ n = 5). For comparison, samples were collected from 22 infection-free volunteers. Since these volunteers resided in the same region and stemmed from similar socioeconomic backgrounds they were classified as endemic normals (EN). Written informed consent was obtained from all individuals. Ethical clearance for this study was given by both the University of Bonn Ethics committee (“Ethikkommission der Medizinischen Fakultät der Rheinischen Friedrich-Wilhelms-Universität Bonn”), the Committee on Human Research Publication and Ethics, University of Science and Technology, Kumasi and the Liverpool School of Tropical Medicine, UK.

### Parasitological assessment

The entire study population was screened for other helminth infections via stool and urine analysis, furthermore subclinical malaria infection was determined using NADAL Test (nal von Minden, Moers, Germany). To verify an ongoing filarial infection patients were tested for circulating filarial antigen (CFA) which was detected using the TropBio® ELISA test kit (TropBio, Townsville, Australia) as described previously [39], [40]. Due to the periodicity of the MF, levels of MF were determined from blood samples collected between 21–23 h. In short, 1 ml of blood was filtered through a 5 µm Whatman Nucleopore filter (Karl Roth, Karlsruhe, Germany), the retained MF were then visualized via Giemsa staining and microscopically counted. 92 individuals were MF^+^ and 67 were MF^−^. 10 individuals (n = 6 MF^+^ and n = 4 MF^−^) were also positive for one other helminth infection (*Ascaris lumbricoides* n = 8, *Strongyloides stercoralis* n = 1, *Trichuris trichiura* n =  1). As mentioned above, 22 volunteers were diagnosed as uninfected controls since they were both CFA and MF negative. Study participants were examined for occurrence of adult filariae and lymph dilation in the scrotum using a portable ultrasound machine (SONOSITE 180 Plus, Sonosite, Bothell, USA) equipped with a 7.5 MHz linear transducer [19]. The number of worm nests was determined by detecting movement of adult worms using the Pulse Wave Doppler mode and all infected patients harbored at least one detectable worm nest (Filarial Dance Sign, FDS) [4]. Dilation of lymphatics and lymphatic vessels at the position of the worm nest and the maximum dilation of a lymphatic vessel (without worms) in the supratesticular area were measured in all participants (n = 181). The latter parameter was then evaluated using the following grading system: no dilation = stage 0; minimal dilation (<0.2 cm) = stage 1; mild dilation (0.21–0.5 cm) = stage 2; moderate dilation (0.51–1.0 cm) = stage 3 and severe dilation (>1.1 cm) = stage 4 [38].

### Antigens and antibodies

A soluble antigenic extract of *Brugia malyi* (*B.m.*) was prepared from adult worms that developed in Mongolian jirds (*Meriones unguiculatus*) as published earlier [41]. Recombinant full-length *Plasmodium falciparum* Merozoite Surface Protein 1 (MSP-1) was prepared as previously described [42]. LPS (*Serratia marescens*) was obtained from Sigma-Aldrich (Munich, Germany). *B.m.* and MSP-1 were tested for their endotoxin levels using the kinetic *Limulus* amoebocyte lysate assay (Charles River, Charleston, SC), the final endotoxin levels in soluble *B.m.* extract or MSP-1 were <0.16EU/ml or <0.05EU/ml respectively. Anti-CD3 and anti-CD28 antibodies were purchased from eBiosciences (Frankfurt, Germany). Secondary antibodies for IgG1-4 were obtained from Sigma-Aldrich, whereas that for IgE was purchased from Southern Biotech (Birmingham, USA).

### PBMC preparation

Venous blood was collected into EDTA-filled monovettes. Blood was transported at 4–8°C from field stations to the local laboratory where PBMCs were directly isolated. In brief, 7 ml of blood was transferred into Ficoll containing Leucosep tubes (Greiner Bio-One, Frickenhausen, Germany) and centrifuged for 20 min at 800× g at room temperature. Plasma layers were removed and frozen until further use. Cell suspensions were then washed twice with sterile PBS (8 min at 400g at room temperature and re-suspended in RPMI 1640 medium (PAA, Linz, Austria), supplemented with 10% FCS (PAA) and 50 µg/ml of gentamicin (PAA), before counting with trypan blue (Sigma-Aldrich, Munich, Germany).

### *In vitro* cell cultures

For re-stimulation, 2×10^5^ PBMCs/well were plated into 96-well plates (U-shaped, Greiner Bio-One). PBMCs were left either unstimulated or stimulated in triplicates with the following stimuli: *B.m.* extract (5 µg/ml), anti-CD3/anti-CD28 (10 µg/ml and 2.5 µg/ml, respectively), MSP-1 (0.25 µg/ml), or LPS (50 ng/ml). Cultures were incubated for 72 hours at 37°C in 5% CO_2_. Supernatants were collected and frozen until further use.

### Determination of Th-like profiles

Culture supernatants from stimulated PBMCs were thawed on ice and analyzed for the content of IL-4, IL-5, IL-6, IL-10, IL-13, IL-17, IFN-γ, TGF-β and TNF using R&D Duo set ELISA (R&D Systems, Wiesbaden-Nordenstadt, Germany) according to the manufacturer's instructions. In brief, ELISA plates (Greiner Bio-One) were coated with 50 µl capture antibody per well overnight. The plates were blocked for 1 hour with 1% BSA in PBS and subsequently incubated for 2 hours with 50 µl/well supernatants or standards. Plates were incubated for 2 hours with 50 µl/well of detection antibody. Thereafter, plates were incubated in the dark with 50 µl/well Streptavidin-horseradish-peroxidase for 20 minutes. Finally, 50 µl/well substrate solution containing TMB [tetramethylbenzidine], (Sigma-Aldrich) were added to the plates and after 30 minutes the reaction was stopped with 25 µl/well 2N H_2_SO_4_ (Sigma-Aldrich). All steps were fulfilled at room temperature. The plates were measured using the SpectraMAX ELISA reader (Molecular Devices, Sunyvale, USA with wavelength correction (450 nm and 570 nm). Data were analyzed with SOFTmax Pro 3.0 software.

### Analysis of immunoglobulins

Plasma from study participants was investigated for immunoglobulins (total IgG, IgG1, IgG2, IgG3, IgG4 and IgE) using the Cytometric Bead Assay Flex Set (Becton Dickinson, Heidelberg, Germany). Plasma was diluted in RPMI 1640 medium (IgG total, 1∶100,000, IgG1, 1∶100,000, IgG2-4, 1∶20,000, IgE, 1∶2,000) and according to the manufacturer's instructions incubated with the appropriate amount of beads for one hour at room temperature. Afterwards, samples were washed with 500 µl washing buffer for five minutes at 400× g and then incubated for two hours in the dark with their appropriate detection antibody. In the last step, samples were washed, re-suspended and solved in 200 µl washing buffer and analyzed by flow cytometry using the FACS-Canto I (BD). Data were analyzed using FCAP-Array software and BD FACSArray Bioanalyzer.

### Assessment of antigen-specific IgE and IgG subclasses

Individual plasma samples were analyzed for levels of antigen-specific IgE and IgG1-4. In brief, 96-well polysorb plates (Nunc, Roskilde, Denmark) were coated overnight at 4°C with 50 µl/well of 5 µg/ml *B.m.* extract in PBS (pH 9.6). Plates were washed three times in 0.05% Tween/PBS (pH 7.2), once in PBS alone and blocked with 200 µl/well of 1% BSA/PBS for two hours at room temperature. After an additional washing step, 50 µl/well of diluted plasma was added in triplicates (1∶500 for specific IgG1-4 and 1∶20 for specific IgE) and incubated overnight at 4°C. After washing, 50 µl/well of the biotinylated secondary antibodies were added for two hours at room temperature, IgG1 (1∶1,000), IgG2 (1∶15,000), IgG3 (1∶4,000), IgG4 (1∶15,000); IgE (1∶1,000). Following the next wash, 50 µl/well streptavidin-peroxidase (Roche Diagnostics, Mannheim, Germany; 1∶5,000) was incubated for 45 minutes. After the final wash, 50 µl/well of substrate solution containing TMB (tetramethylbenzidine, Sigma-Aldrich, Munich, Germany) were used and the reaction was stopped with 25 µl/well 2N H_2_SO_4_. The plates were measured as mentioned above. Pooled plasma from ten patients was used for the generation of calibration curves and assigned arbitrary units for the specific anti-filarial antibodies. In plasma from healthy European donors helminth-specific IgG1-4 and IgE were not detectable (data not shown).

### Statistical analysis

Statistical analyses were performed using the software SPSS version 19 (SPSS Schweiz Ag, Zurich, Switzerland), the PRISM 5 programme (GraphPad Software, Inc., La Jolla, USA) and SAS version 9.2 (SAS Institute Inc. Cary, NC, USA). To compare the three groups a Kruskal-Wallis-test was performed and, if significant, followed by Mann-Whitney–U test for the comparison of two groups. P values of 0.05 or less were considered significant. Where indicated the Cochran-Armitage test was used to determine a trend. In addition, data were assessed using a generalized linear model analysis using age as a covariate.

## Results

### Clinical evaluation of lymphatic filariasis patients shows an increased number of worm nests in MF^+^ individuals

**Figure 1:**
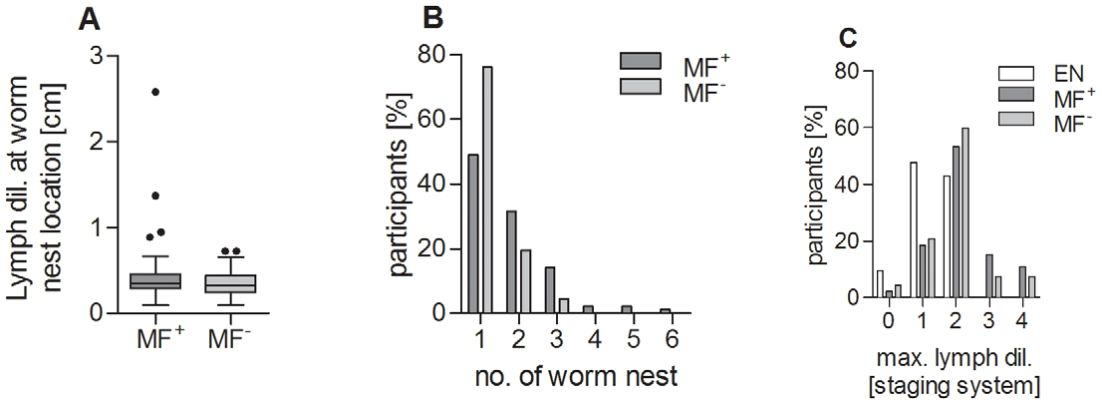
Active circulation of MF is associated with the number of worm nests. Using ultrasound, infected individuals were examined for (A) the lymph dilation around the worm nests and (B) for the amount of worm nests (FDS). In addition, patients were also examined for (C) the maximum degree of their supratesticular lymph dilation, presented as a staging system. Box whisker plots in (A) show median, interquartile ranges and outliers of individually tested patients. Bars in (B) and (C) show the number of individuals and their correlating amount of worm nests or lymph dilation respectively. Data in (B) were tested for significance with the Cochran-Armitage trend test (p<0.001).

Our study cohort comprised of 159 *W. bancrofti*-infected male patients and 22 endemic normal volunteers (EN) from 25 villages in Ghana. Amongst the infected individuals, 92 were microfilaremic (MF^+^) and 67 amicrofilaremic (MF^−^) and the age distribution within the groups was similar (Figure S1). Patients were selected, as part of a larger treatment study, on the basis of adult worms (FDS^+^) and CFA^+^ (Table 1). Patients recruited to the study were negative for lymphedema (LE). Within the MF^+^ and MF^−^ groups no significant differences could be observed in lymph dilation measurements monitored at worm nest locations (Figure 1A). MF^+^ patients did however possess significantly more scrotal worm nests when compared to MF^−^ patients (Figure 1B), which was confirmed using the Cochran-Armitage trend test (p<0.001). Although there was a slight positive correlation between the number of worm nests and the amount of circulating microfilaria (Spearman r = 0.32, p<0.001), there was no correlation with either the age of the individual or the villages in which they were residing. With regards to CFA, we also found a positive correlation (r = 0.68, p<0.001) between the concentration of CFA and MF. Whereas Figure 1A illustrates the local lymph dilation around the detected worm nests, Figure 1C depicts the maximal detectable lymph dilation within the whole scrotal tissue, including the area of the spermatic cord which is not necessarily co-localized with the position of worm nests. Here, there was no significant difference between the two *W. bancrofti*-infected groups. In EN, maximal dilation of scrotal lymphatic vessels was also assessed and they displayed lymph dilation between grades 0 and 2 (Figure 1C). Participants had participated in MDA with an average of two rounds anti-filarial therapy (IVM and ALB), the most recent round being ≥10 months before start of the study (Table 1). Previous studies have documented that following such therapy MF reappear in 77% of infected patients after 12 months [43]. Thus, we conclude that our asymptomatic amicrofilaremic participants presented latent infection.

**Table 1:** Characteristics of study population.

	MF^+^ (92)	MF^−^ (67)	EN (22)
age	34.68 (18–50)	34.61 (18–50)	32.50 (19–45)
FDS	positive	positive	negative
CFA	positive	positive	negative
Rounds of ivermectin	2.17	2.09	n.d.
Number of scrotal worm nests	1.80	1.28	0
m-mode average (cm)	0.41	0.36	0
MF count (MF/ml)	979.16 (1–7,590)	0	0

Age: mean (range); rounds of ivermectin: mean; Number of scrotal worm nests: mean; m-mode: mean; MF count: mean (range).

### Filarial-specific IL-5 responses are elevated in latently infected individuals

**Figure 2:**
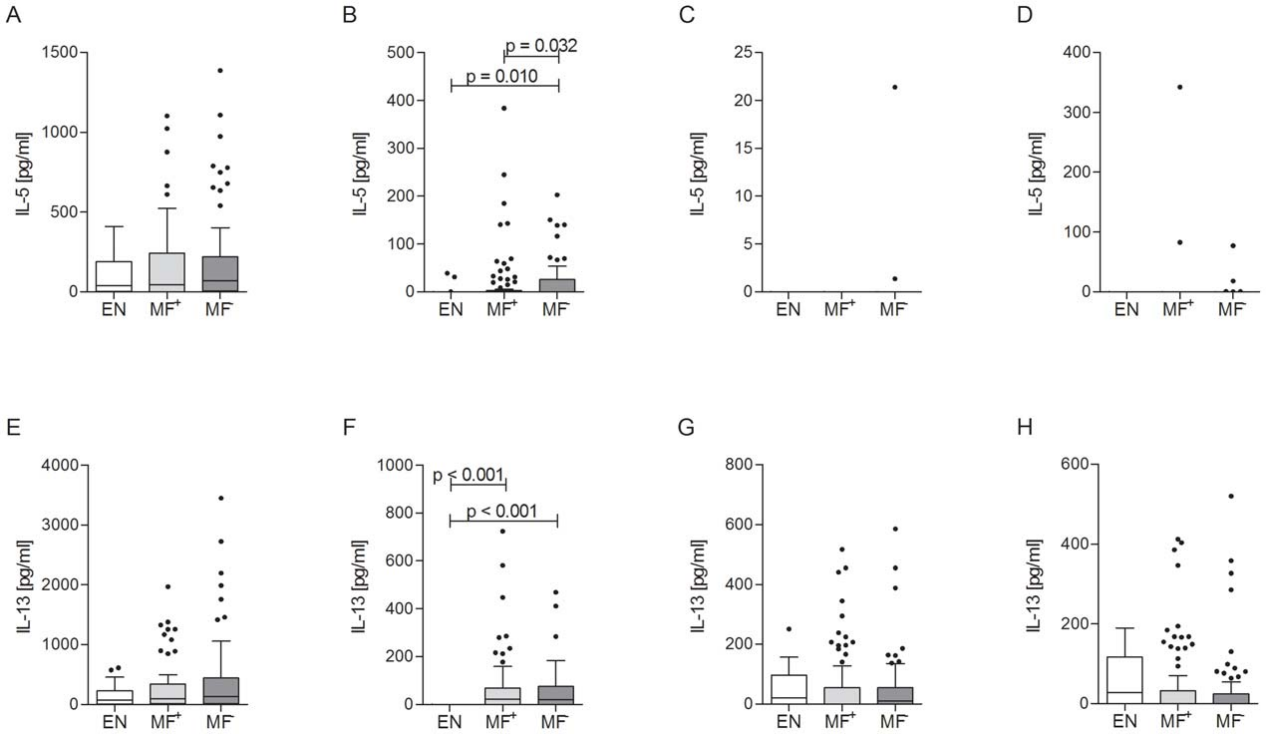
Patent infection alters filarial-specific Th2-like responses. Isolated PBMCs (2×10^5^/well) from endemic controls (EN) or filarial-infected MF^+^ or MF^−^ patients were stimulated with either anti-CD3/anti-CD28 (10 µg/ml/2.5 µg/ml; A and E), *Brugia malayi* extract (*B.m.*, 5 µg/ml; B and F), MSP-1 0.25 µg/ml; C and G) or LPS (50 ng/ml; D and H) for 72 hours. Thereafter, levels of IL-5 (A–D) or IL-13 (E–H) were measured in the culture supernatants via ELISA. Graphs show box whiskers with median, interquartile ranges and outliers after background subtraction. Statistical significances between the indicated groups were obtained after Kruskal-Wallis and Mann-Whitney-U tests.

Although much is known about the immunological differences between asymptomatic MF^+^ patients and those with chronic pathology, little is known about the differences in antigen-specific responses of asymptomatic patent and latent individuals. To address the immunological profile of MF^+^ and MF^−^ patients, PBMCs were isolated from infected individuals and EN and stimulated with anti-CD3/anti-CD28, *B.m.* extract, MSP-1 and LPS for three days. Th2-like profiles were determined on levels of IL-5 (Figure 2A–D), IL-13 (Figure 2E–H) and IL-4 (data not shown) in the supernatant. IL-5 and IL-13 are typical hallmarks for helminth infections and Figures 2A and E show that upon unspecific T cell activation, both groups of infected individuals and EN produced similar levels of these cytokines. With regards to filarial-specific responses, PBMCs of MF^−^ patients made significantly more IL-5 than MF^+^ individuals (Figure 2B), whereas EN hardly responded to this antigen at all. As shown earlier by Limaye *et al.* [44], when correlating antigen-specific IL-5 production with the percentage of blood-derived eosinophils, no significant differences could be observed for either infected group (data not shown). IL-13 was strongly produced by all infected individuals with no significant differences between MF^+^ and MF^−^ groups. When compared to EN however, both infected groups produced significantly higher levels of IL-13 (Figure 2F). In response to MSP-1 and LPS, we could not detect any significant differences between the two infection groups (Figures 2 C, D, G and H). IL-4 production was not detected in any of the stimulations (data not shown).

### Amicrofilaremic patients present elevated IL-17 responses

**Figure 3 pntd-0001611-g003:**
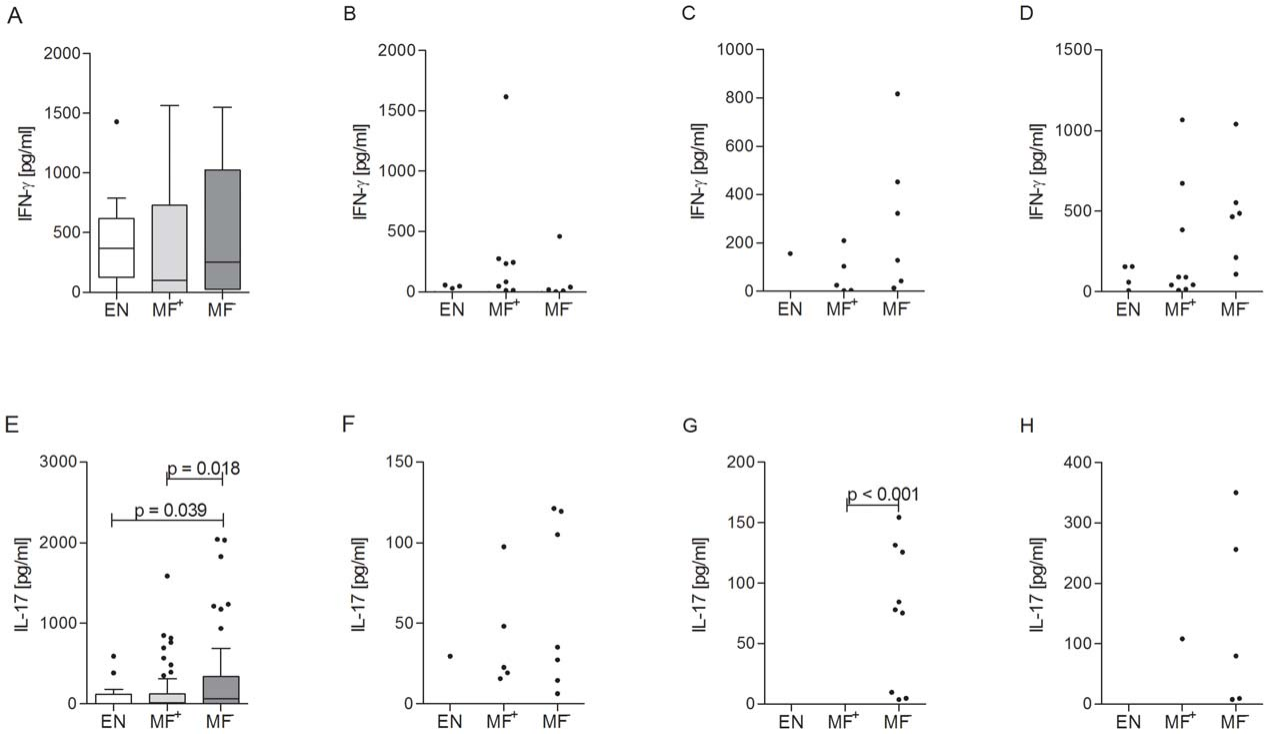
Latent infected patients show elevated IL-17 but not Th1-like responses. Isolated PBMCs (2×10^5^/well) from endemic controls (EN) or MF^+^ or MF^−^ patients suffering from LF were stimulated with either anti-CD3/anti-CD28 (10 µg/ml/2.5 µg/ml; A and E), *Brugia malayi* extract (*B.m.* 5 µg/ml; B and F), MSP-1 (0.25 µg/ml; C and G) or LPS (50 ng/ml; D and H) for 72 hours. Thereafter, levels of IFN-γ (A–D) or IL-17 (E–H) were measured in the culture supernatants via ELISA. Graphs show box whiskers with median, interquartile ranges and outliers after background subtraction. Statistical significances between the indicated groups were obtained after Kruskal-Wallis and Mann-Whitney-U tests.

To observe whether there were any diversities in the Th1- and Th17-like profiles of microfilaremic and amicrofilaremic patients we also measured the secreted levels of IFN-γ (Figure 3A–D) and IL-17 (Figure 3E–H). PBMC from only a limited number of patients secreted these cytokines upon re-stimulation with *B.m.* extract (Figures 3B and F). Similarly, few responders were also observed upon stimulation with MSP-1 (Figures 3C and G) and LPS (Figures 3D and H). Indeed, only MF^−^ patients responded to MSP-1 in terms of IL-17 production. These responders did not correlate with those patients that had a subclinical plasmodial infection. Albeit only in a small portion of the study cohort (19/181), further statistical analysis revealed that these subclinical malaria patients did not differ in any of their responses when compared to the malaria negative patients. Interestingly, when compared to patently infected patients and EN, the MF^−^ group also showed significantly elevated IL-17 responses upon anti-CD3/anti-CD28 stimulation (Figure 3E). Although non-significant, after stimulation with anti-CD3/anti-CD28 the median IFN-γ response of MF^+^ patients was lower than either of the other two groups (Figure 3A). These results indicate that neither infected group displayed a dominant filarial-specific Th1-like response.

### Filarial-specific IL-10 is enhanced in latently infected individuals

**Figure 4 pntd-0001611-g004:**
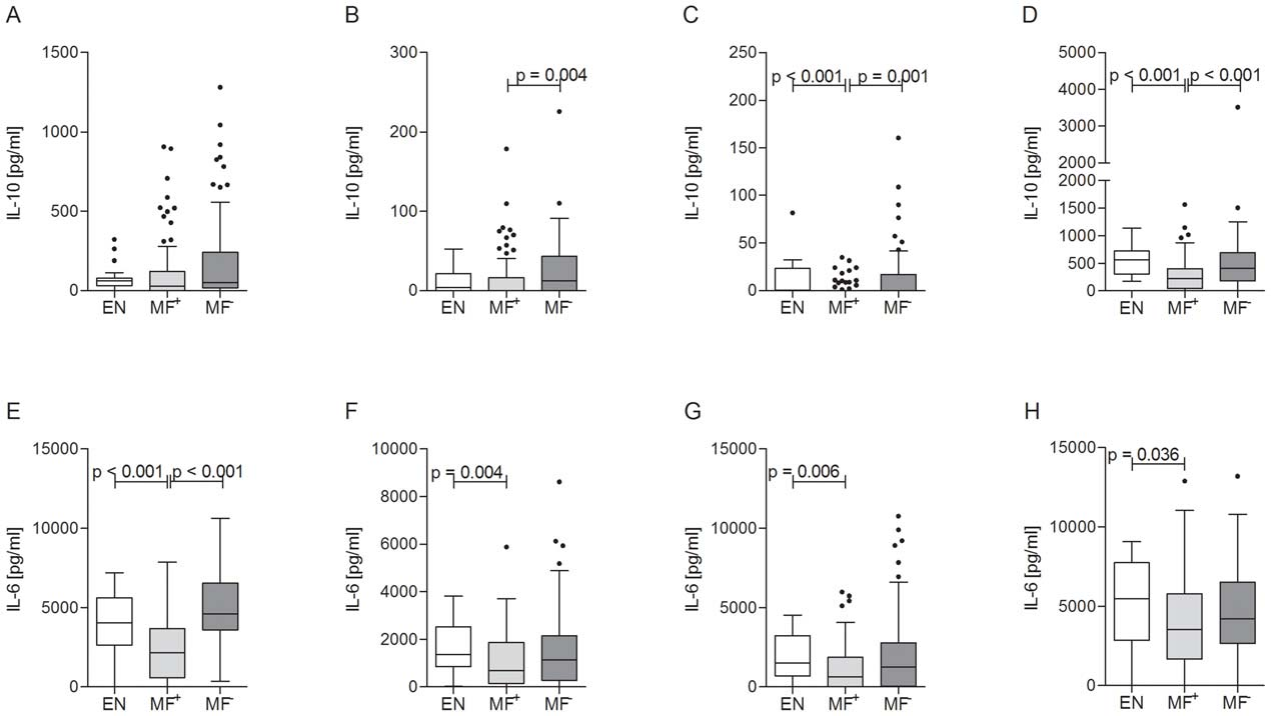
Regulatory responses are enhanced in latently infected individuals. Isolated PBMCs from endemic controls (EN) or patients infected with *W. bancrofti* were stimulated with anti-CD3/anti-CD28 (10 µg/ml/2.5 µg/ml; A, E), *Brugia malayi* extract (5 µg/ml, B, F), MSP-1 (0.25 µg/ml; C, G) or LPS (50 ng/ml, D, H) for 72 hours. Thereafter, culture supernatant was tested via ELISA for cytokine release of IL-10 and IL-6. Graphs show box whiskers with median, interquartile ranges and outliers after background subtraction. Statistical significances between the indicated groups were obtained after Kruskal-Wallis and Mann-Whitney-U tests.

Next, using the readout of IL-10 production we analyzed the regulatory responses of these patients. We found that in our patient cohort, all IL-10 responses from MF^+^ patients were lower than those from MF^−^ patients irrespective of the applied stimulus. These included the responses to malaria peptide (Figure 4C) and LPS (Figure 4D) which were significantly lower when compared to responses from EN and MF^−^ patients. These data imply that MF are able to immunomodulate responses to other parasitic antigens such as those derived from malaria species as well as to a bacterial stimulus. Due to the high background production of TGF-β, no differences could be determined within the different groups, irrespective of antigen stimulus (data not shown). Finally, we observed a general down regulation of IL-6 release from PBMCs in the microfilaremic group when compared to uninfected individuals and this was irrespective to the applied stimulus (Figures 4E–H). In the case of T cell activation we also detected a significant suppression of IL-6 release from PBMCs of patently infected individuals (Figure 4E), again demonstrating down-regulated responses in these individuals.

### Microfilaremic individuals show dampened TNF responses

**Figure 5 pntd-0001611-g005:**
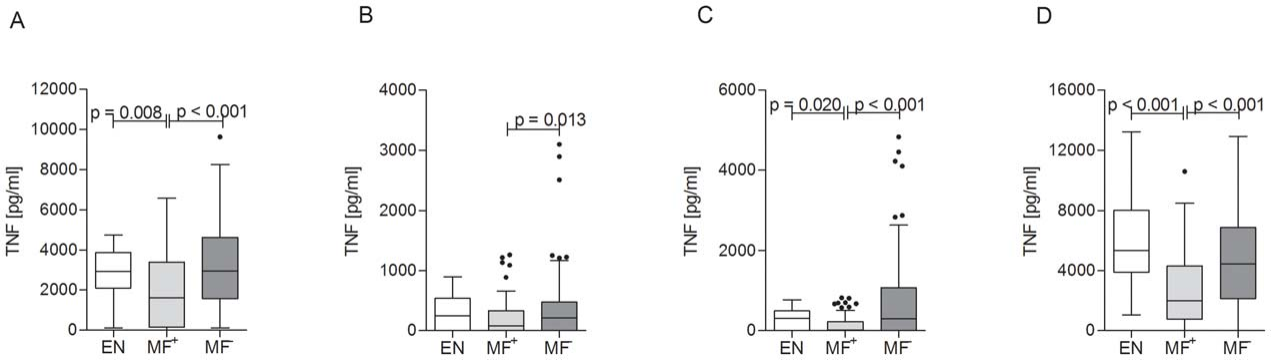
Circulating microfilariae dampen release of TNF. Isolated PBMCs from endemic controls (EN) or patients suffering from LF were stimulated with anti-CD3/anti-CD28 (10 µg/ml/2.5 µg/ml; A), *Brugia malayi* extract (5 µg/ml; B), LPS (50 ng/ml; C) or with MSP-1 (0.25 µg/ml; D) for 72 hours. Thereafter, culture supernatant was tested via ELISA for cytokine release of TNF. Graphs show box whiskers with median, interquartile ranges and outliers after background subtraction. Statistical significances between the indicated groups were obtained after Kruskal-Wallis and Mann-Whitney-U tests.

Next, stimulated PBMCs were investigated for their TNF production since during filariasis this cytokine has been shown to be a feature of acute infection [45]. Strikingly, after stimulation with anti-CD3/anti-CD28, *B.m.* extract, MSP-1 or LPS we found significantly suppressed TNF responses from MF^+^ patients when compared to either EN or the latent infected group (Figure 5A, B, C and D). These data highlight that by dampening TNF responses, whether adaptive or innate, there is a potential benefit for the helminth since reduced responses to MF would enhance the chances for transmission. In association, the higher levels of TNF in latent individuals may indicate a possible mechanism in suppressing MF release or even rapid destruction of MF.

### Quantitative assessment of IgG and IgE levels

**Figure 6 pntd-0001611-g006:**
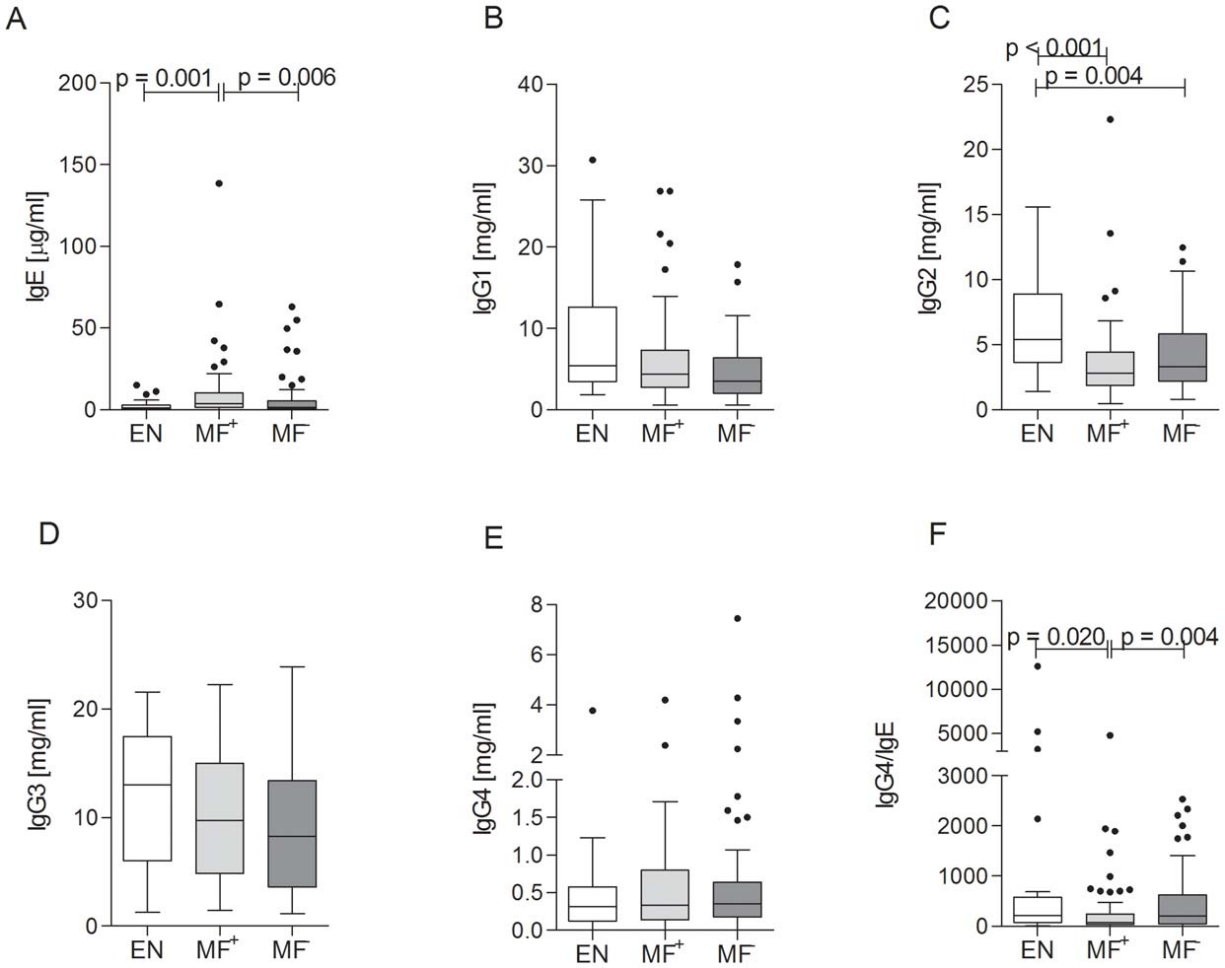
Patently infected individuals present elevated levels of total IgE. Plasma from all three groups was analyzed for the production of total IgG1 (A), IgG2 (B), IgG3 (C), IgG4 (D) and IgE (E) with the Cytometric Bead Array. (F) shows the ratio of IgG4 and IgE. Graphs show box whiskers with median, interquartile ranges and outliers. Statistical significances between the indicated groups were obtained after Kruskal-Wallis and Mann-Whitney-U tests.

Alongside IL-5, elevated levels of IgE are a hallmark of helminth infection [33]. Therefore, we measured the concentrations of total IgE and IgG subclasses in the plasma of all study participants and uninfected volunteers (Figures 6 A–E). Microfilaremic patients had significantly more IgE when compared to EN (Figure 6A). Interestingly, these levels were also more pronounced when compared to the latent infected group (Figure 6A). With regards to the IgG subclasses, no differences in levels of total IgG1 and IgG3 could be observed between individuals in any of the study groups (Figure 6B and D). However, both patient groups showed a significant reduction of IgG2 when compared to EN (Figure 6C). Although no differences in the levels of IgG4 could be observed between the different cohorts (Figure 6E) the ratio of IgG4 and IgE was significantly reduced in the microfilaremic group when compared to MF^−^ and EN individuals (Figure 6F).

### MF^+^ patients display a predominant antigen-specific IgG4 phenotype

**Figure 7:**
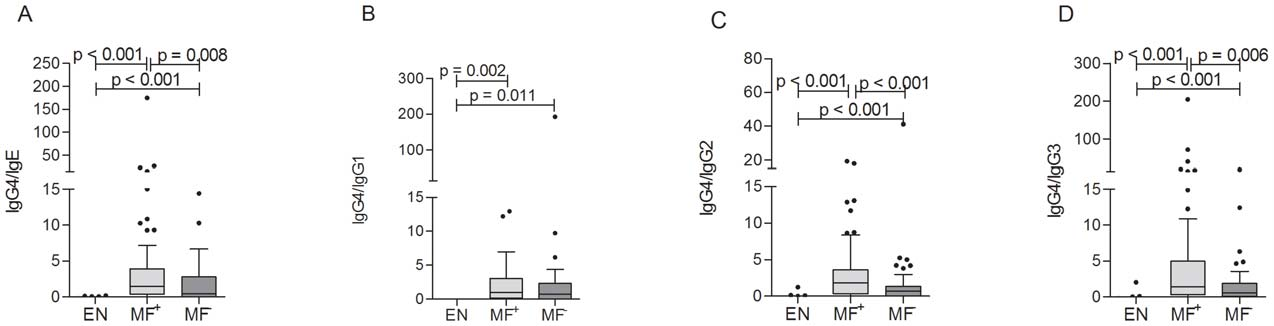
Patent infected patients produce more filarial-specific IgG4. Plasma from all participants was investigated for the presence of helminth-specific immunoglobulins by ELISA. Plates were coated overnight with 5 µg/ml *B.m.* extract in PBS (pH = 9.6), incubated with patient and control plasma overnight and analyzed for specific IgG1-4 and IgE. Presented are the ratios of antigen-specific antibodies IgG4/IgE (A), IgG4/IgG1 (B), IgG4/IgG2 (C) and IgG4/IgG3 (D). Graphs show box whiskers with median, interquartile ranges and outliers. Statistical significances between the indicated groups were obtained after Kruskal-Wallis and Mann-Whitney-U tests.

Finally, individual patients were analyzed for their filarial-specific IgG and IgE expression and results are displayed as ratios of antigen-specific IgG4 to the other isotypes (Figure 7 A–D). As expected, the control group produced only background levels of specific filarial antibodies (Figure 7A–D). Ratios of antigen-specific IgG4/IgE (Figure 7A), IgG4/IgG1 (Figure 7B), IgG4/IgG2 (Figure 7C) and IgG4/IgG3 (Figure 7D) in the infected groups were all significantly higher when compared to the EN group. In contrast to the data generated on the presence of total IgE and IgG4 (Figure 6F), MF^+^ patients showed a dominant expression of filarial-specific IgG4 (Figure 7A). Indeed, this strong IgG4 phenotype in MF^+^ patients was reflected in all ratios (Figures 7A–D). Significant differences between MF^+^ and MF^−^ groups were found in the ratios of IgG4/IgE (Figure 7A), IgG4/IgG2 (Figure 7C) and IgG4/IgG3 (Figure 7D). In summary, patently infected individuals display a strong expression of filarial-specific IgG4 indicating that circulating MF also influence B cell responses.

### Elevated immune responses of MF^−^ patients are independent of age

Previous publications have shown that age may play a role with regards to cytokine production during filariasis [46] and interestingly we also found that an increase in age correlated with increased immune responses. Of note, age distribution was equal between the infected groups (Figure S1). Therefore, we performed regression analysis using age as a covariate and log transformed [log(concentration+0.5)] all data to correct for skewness [47], [48]. Statistically however, this did not change our overall results, that is, PBMCs from latently infected individuals produced more cytokines upon re-stimulation with various stimuli and also the results for the immunoglobulin responses stayed the same. Moreover, upon age correction, additional significant differences become apparent in the scenario of IL-10 production following anti-CD3/anti-CD28 stimulation. Indeed, MF^+^ patients produced significantly less IL-10 when compared to MF^−^ individuals (p = 0.0047) which correlates to the statistical responses observed with the other stimuli (Figure 4B–D). The only variable for which we found an interaction between age and MF status was total IgG3 secretion: here increases in age correlated with elevated IgG3 in MF^−^ but less IgG3 in MF^+^ patients but the latter group still had more IgG3. In addition, we also observed the effect of removing our 12 clinical hydrocele patients and interestingly, all statistical outcomes remained the same as shown in Figures 2–7 with the exception of TNF secretion upon *B.m.* specific re-stimulation. Here however, the trend remained. These data strengthen and confirm other studies which have revealed that it is the parasitic status and not the clinical condition of the individual that influences the observed immunological responses [49].

## Discussion

Helminths are renowned for their ability to immunomodulate the host's immune system to ensure both reproduction and long-term survival and the filarial nematodes are no exception. Several reports have deciphered the immunological status of MF^+^ patients with those presenting chronic pathology and it appears that the balance between Th1/Th2, Treg and immunoglobulin responses, alongside genetic factors of both the parasite and host, may define whether the infected individual is asymptomatic or develops lymphedema [28]. However, little has been reported about the strength and character of immune responses elicited in asymptomatic amicrofilaremic individuals, a cohort of patients which are epidemiologically interesting since they are a dead end for the parasite's life cycle with regards to transmission. To address this aspect, we studied a cohort of non-lymphedema *W. bancrofti*-infected individuals since conclusive differentiation of the infection status can now be readily achieved using various diagnostic tools [6], [7]. Our data shows that in contrast to amicrofilaremic individuals, patients with a patent infection have suppressed responses, which indicates that the parasite actively prevents unwarranted reactions to the transmission stage of the parasite. In addition, these findings indicate that latent individuals have developed immune mechanisms to prevent the development of microfilariae, or MF reaching the peripheral blood (which is the normal night-time scenario in all individuals with periodic LF). Although our patients presented no LE a small proportion had clinical hydrocele in accordance to Mand *et al.*, [38]. Remarkably, the presence of hydrocele did not influence the outcome of dampened responses in the patently infected group, indicating that these two separate pathological outcomes can influence the immune responses in a different manner.

Naturally, the dampening of not only antigen-specific responses but those to bystander antigens would greatly enhance the parasite's chance of survival. Support of this hypothesis was clearly observed by the secretion of TNF, since responses of PBMC isolated from patently infected individuals were strongly down-regulated regardless of the stimulus. In recent years, there have been various reports describing the involvement of TNF during filariasis [45], [50], [51]. However, the majority of these studies have investigated differences between infected and EN or between infected and chronic pathology or between MF^+^ patients and those with episodes of acute filariasis (adenolymphangitis). For example, in studies of acute filariasis, individuals had elevated levels of TNF in their sera when compared to MF^+^ patients [50]. In another study, that compared EN with MF^+^ patients, TNF responses from PBMCs of microfilaremic patients were again dampened if their cells were stimulated with either live L3, live MF or filarial antigen, although the latter was not significant [51]. Reduced levels of TNF were also observed in asymptomatic patients (microfilaremia not determined) when compared to those with chronic pathology [31]. Thus, in correlation with the findings here the presence of circulating MF dampens TNF responses regardless of whether these are antigen-specific, bystander or innate. Intriguingly, *in vitro* studies have implicated that *Wolbachia* endobacteria, but not the worm itself, can elicit TNF secretion from innate host cells in a TLR-dependent manner [15], [16]. Moreover, persistent exposure to *Wolbachia* but not bacteria-free nematode extracts, drives homologous and heterologous tolerance of macrophages to TLR and CD40 ligands and protects against endotoxin shock *in vivo* [52]. Other studies have linked TNF with promoting the production of vascular endothelial growth factors such as VEGF-C and VEGF-A [53], [54], which reportedly contribute to the development of lymphodema and hydrocele [20], [40]. In addition to the decreased secretion of TNF from PBMCs of patently infected individuals we also observed suppressed IL-17 responses. Interestingly, *in vitro*, rIL-17 can drive TNF production in macrophages [55] and a combination of rIL-17 and rTNF was shown to increase the production of angiogenic factors like VEGF, from fibroblasts [56]. Since we also observed differences in IL-6 production between MF^+^ and amicrofilaremic individuals and it is well known that human IL-17 is induced by IL-6 in combination with TGF-β [57], a hypothetical scenario begins to emerge: worm death could promote the release of *Wolbachia* which in turn elicits TNF production by triggering TLR [58]. Enhanced TNF, and possibly IL-6, coupled to stronger IL-17 responses could instigate the production of VEGF and promote the onset of pathology. In association, studies revolving around the immune responses of chronic pathology patients observed elevated levels of IL-17 and TNF [31]. The presence of this cytokine combination in our latent individuals may reflect a higher susceptibility to pathology especially since a cross-sectional study has indicated that the onset of chronic pathology is linked to amicrofilaremic status [59]. Theoretically, the normal release of MF could counterbalance this immunological milieu circumventing unwarranted responses.

Interestingly, unlike TNF, secretion of IFN-γ was not detectable for any of the tested stimuli except direct T cell activation (Figure 3A), which showed that microfilaremic patients produced less IFN-γ, albeit insignificantly, than latent infected individuals. In association, other studies comparing MF^+^ individuals and those presenting chronic pathology showed that there were no significant differences in the numbers of polyclonally activated Th1 cells [28], [31]. With regards to Th2-like responses, all infected patients produced more IL-5 and IL-13 than the EN upon co-culture with the helminth antigen. Although a previous study was unable to observe significant increases of IL-5 expression levels in MF^−^ individuals [60], we observed elevated IL-5 responses in amicrofilaremic patients upon filarial-specific re-stimulation. Interestingly, this reflects studies performed with the murine model of filariasis which have revealed that in the absence of IL-5 infected mice present higher and longer microfilaremic burdens when compared to wildtype strains [61]. Although we could not confirm a negative correlation between the amount of MF and secreted levels of IL-5, as described for *Onchocerca volvulus* infection [62], we could show that microfilaremic individuals produced less IL-5 and that MF levels were correlated to the number of worm nests.

It is generally accepted that during filariasis, IL-10 and TGF-β play an immunosuppressive role but the source of IL-10 remains inconclusive [63], [64], [65], [66]. In onchocerciasis, the presence of IL-10 is clearly associated with a protective immunological scenario: high IgG4, IL-10 and Treg are associated with low pathology whereas high levels of IgE, IL-4 and eosinophilia are common in patients with severe pathology [9], [22], [64], [67]. In contrast to latent individuals, we found elevated polyclonal IgE levels in MF^+^ individuals (Figure 6A). Such high levels of IgE may facilitate the persistence of infection through the production of irrelevant (not parasite Ag-specific) immunoglobulins, that saturate high affinity IgE receptors expressed on mast cells which renders them unable to be specifically cross-linked by parasite antigen [28]. Interestingly, although filarial-specific IL-5 and IL-10 responses were actually dampened in the presence of microfilaria, the ratio of antigen-specific IgG4 to IgE became elevated. Since IgG4 and IgE compete for the same binding sites, determining the ratio provides an indication about the pathophysiological conditions, that is, whether the antigen triggers an IgE mediated hypersensitivity response or whether this response can be blocked by IgG4 [68]. The quantities of specific IgG4 produced in filariasis patients can be remarkably high. In patients presenting elephantiasis, IgG4 levels are similar to those found in EN (57% and 55% of filarial-specific IgG respectively) whereas in MF^+^ carriers the amount is significantly higher (88%) [33]. Upon analyzing the ratio of specific IgG4 to IgE and IgG subclasses, we detected a strong dominance of IgG4 in the microfilaremic group. With regards to IgE, this confirms a study performed by Jaoko *et al.* in East Africa and interestingly, this group determined that specific antibodies levels reflect infection status rather than chronic lymphatic disease [49].

In summary, upon comparison with MF^+^ individuals, amicrofilaremic patients display an increased profile of TNF, IL-17, IL-10, IL-6 and filarial specific Th2-like responses. Since age has been shown to play a role in filarial-specific immune responses, we used regression analysis to assess the influence of age on our data. In the majority of our stimulation scenarios, we found that an increase in age did correlate with increased cytokine production. However, age did not alter the overall outcome, that is, dampened responses shown by the MF^+^ patients, indicating that is the infection status which determines the immune profile. This elevated cytokine milieu, both filarial and bystander specific, may contribute to the induction of other immunological pathways such as VEGF which in turn promotes overt pathology. In contrast, the immunosuppressive pattern in MF^+^ patients is complemented by the high ratio of specific IgG4/IgE. The prevalence of IgG4 provides protection to both host and parasite since it provides a mechanism in order to counter-regulate high IgE and thus avoid excessive immunopathology. Interestingly we saw the described difference between the two infected groups, which did not suffer from chronic pathology, indicating again that the presence of microfilaria seems to be relevant to induce immunosuppression. Such tempered immune responses would provide an environment that benefits the transmission phase of the parasite. The findings herein provide novel insight into the immunology of asymptomatic amicrofilaremic individuals, a previously neglected cohort of patients. Their elevated immune responses may provide the key into elucidating alternative therapeutic treatments which would essentially block transmission and consequently eradicate the infection.

## Supporting Information

Figure S1 **Equal age distribution amongst filarial-infected individuals.** Since age has been reported to play role in filarial-induced immune responses we assessed the age distribution amongst our infected males. No significant differences could be observed. Further details of the patients groups can be observed in Table 1. Symbols represent individuals with each group MF^+^ (n = 92) and MF^−^ (n = 67).
(TIF)
