# Supplementary figures and images for: Elevated Adaptive Immune Responses Are Associated with Latent Infections of Wuchereria bancrofti
Source: PLoS Negl Trop Dis. 2012 Apr 3;6(4):e1611. doi: 10.1371/journal.pntd.0001611 (PMC3317915; doi:10.1371/journal.pntd.0001611)

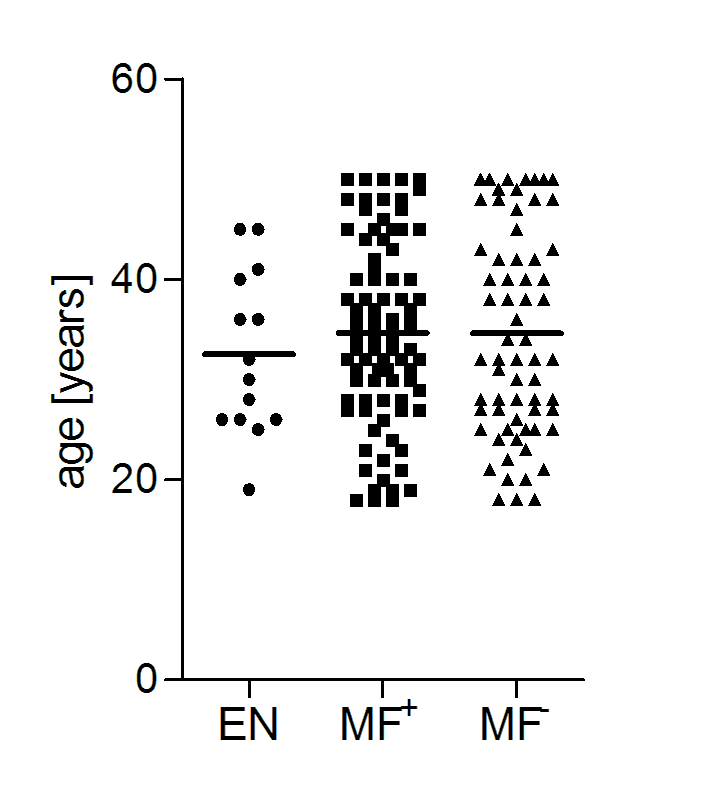

Supplement: Figure S1 — Equal age distribution amongst filarial-infected individuals. Since age has been reported to play role in filarial-induced immune responses we assessed the age distribution amongst our infected males. No significant differences could be observed. Further details of the patients groups can be observed in Table 1. Symbols represent individuals with each group MF+ (n = 92) and MF− (n = 67). (TIF) [file pntd.0001611.s001.tif]
